# Supplementary material for: Highly Sensitive and Stable Multifunctional Self-Powered Triboelectric Sensor Utilizing Mo2CTx/PDMS Composite Film for Pressure Sensing and Non-Contact Sensing
Source: Nanomaterials (Basel). 2024 Feb 27;14(5):428. doi: 10.3390/nano14050428 (PMC10934654; doi:10.3390/nano14050428)
Supplement: Supplementary file 1 [file nanomaterials-14-00428-s001.zip › nanomaterials-2873009-supplementary.pdf]

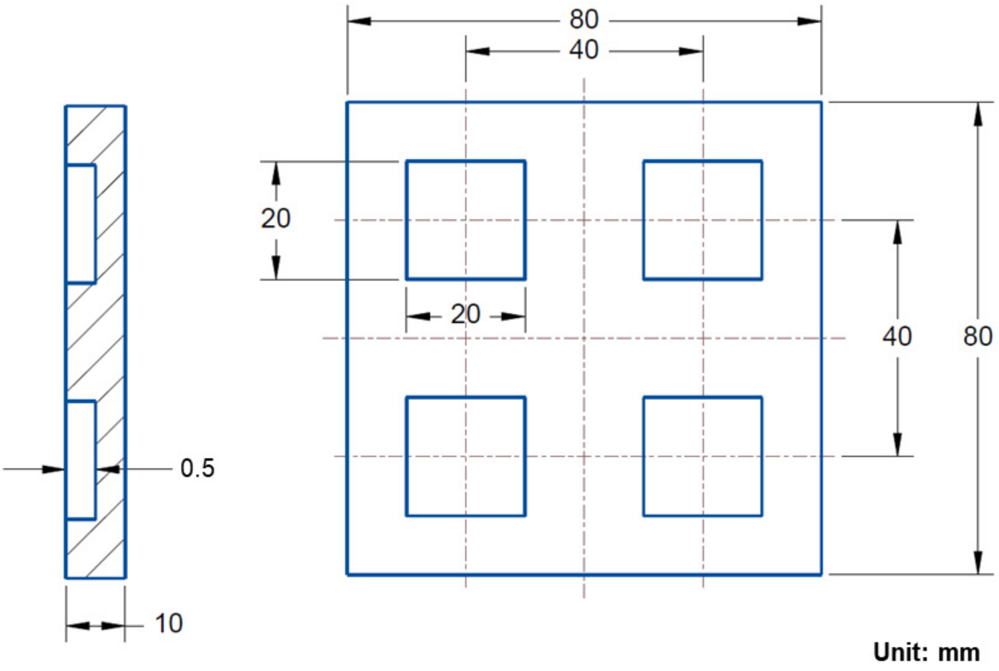

Figure S1. Dimensional drawing of PTFE mould

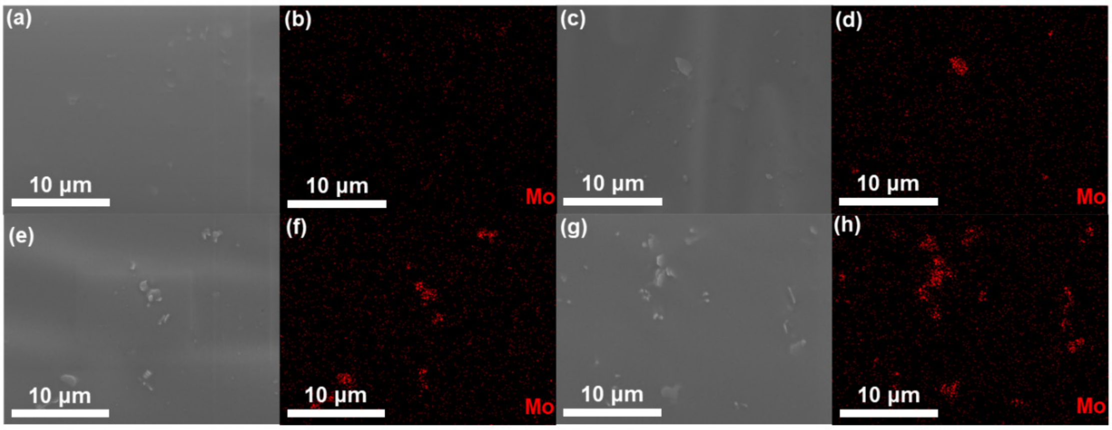

Figure S2. The EDS images depict Mo_2CT_x particles that have been mixed with varying mass fractions.: (a-b) The EDS images of Mo_2CT_x/PDMS composite films mixed with Mo_2CTx particles with a mass fraction of 1 wt%. (c-d) The EDS images of Mo_2CT_x/PDMS composite films mixed with Mo_2CT_x particles with a mass fraction of 2 wt%. particles mixed. (e-f) The EDS images of Mo_2CT_x/PDMS composite films mixed with Mo_2CT_x particles with a mass fraction of 4 wt%. (g-h) The EDS images of Mo_2CT_x/PDMS composite films mixed with Mo_2CT_x particles with a mass fraction of 5 wt%.

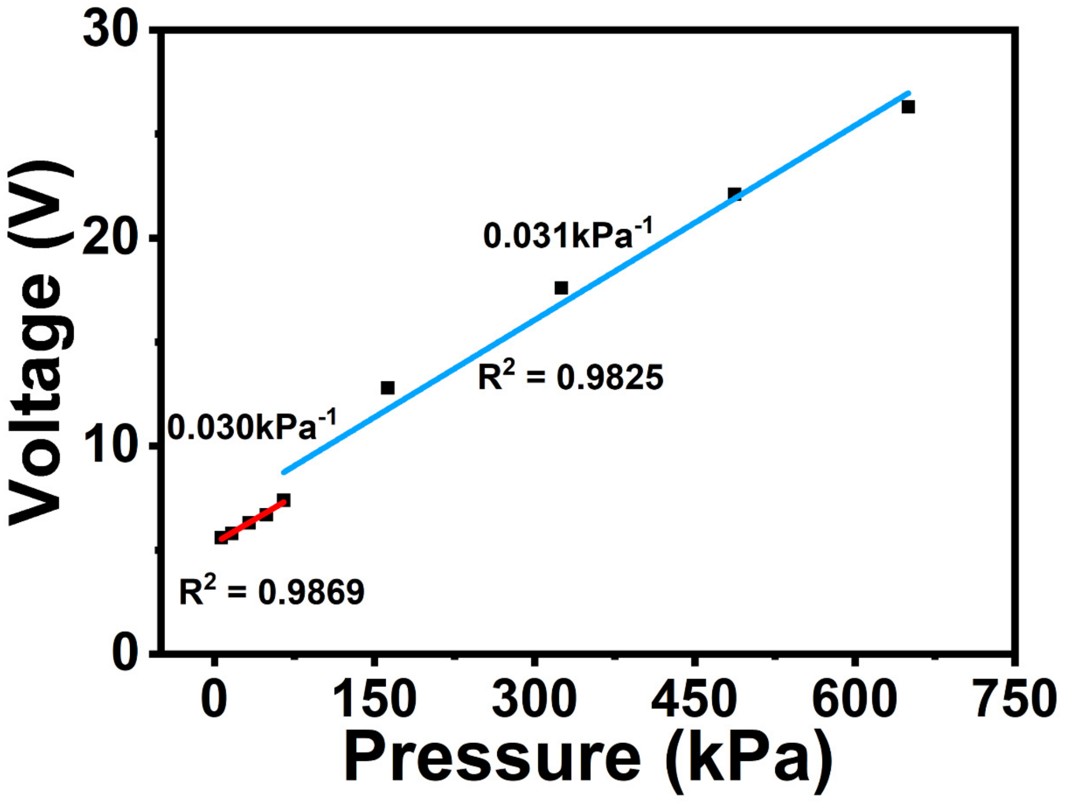

Figure S3: The output voltage and pressure relationship curve of Mo-TES0.

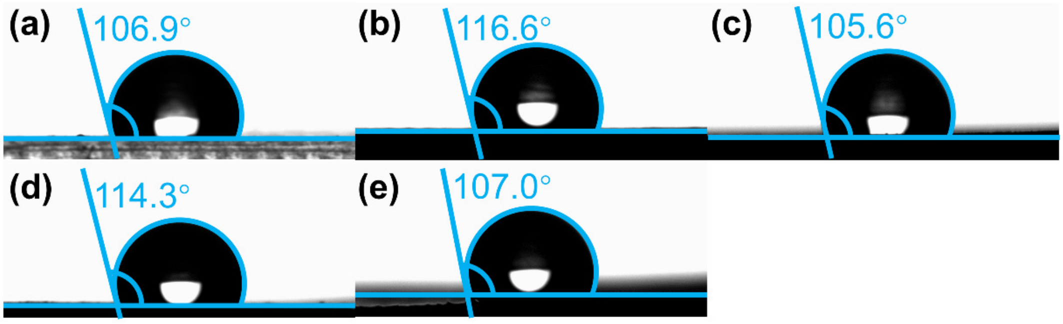

Figure S4. The water contact angle of Mo_2CT_x particles mixed with varying mass fractions is depicted in the images: (a) The water contact angle images of pure PDMS films. (b) The water contact angle images of Mo_2CT_x/PDMS composite films mixed with Mo_2CT_x particles with a mass fraction of 1 wt%. (c) The water contact angle images of Mo_2CT_x/PDMS composite films mixed with Mo_2CT_x particles with a mass fraction of 2 wt%. (d) The water contact angle images of Mo_2CT_x/PDMS composite films mixed with Mo_2CT_x particles with a mass fraction of 4 wt%. (e) The water contact angle images of Mo_2CT_x/PDMS composite films mixed with Mo_2CT_x particles with a mass fraction of 5 wt%.
